# Characterization of an In Vitro/Ex Vivo Mucoadhesiveness Measurement Method of PVA Films

**DOI:** 10.3390/polym14235146

**Published:** 2022-11-26

**Authors:** Laura Müller, Christoph Rosenbaum, Julius Krause, Werner Weitschies

**Affiliations:** Department of Biopharmaceutics and Pharmaceutical Technology, Institute of Pharmacy, University of Greifswald, Felix-Hausdorff-Str. 3, 17489 Greifswald, Germany

**Keywords:** in vitro–ex vivo correlation, mucoadhesive films, tensile studies, porcine small intestine

## Abstract

Transmucosal drug delivery systems can be an attractive alternative to conventional oral dosage forms such as tablets. There are numerous in vitro methods to estimate the behavior of mucoadhesive dosage forms in vivo. In this work, a tensile test system was used to measure the mucoadhesion of polyvinyl alcohol films. An in vitro screening of potential influencing variables was performed on biomimetic agar/mucin gels. Among the test device-specific factors, contact time and withdrawal speed were identified as influencing parameters. In addition, influencing factors such as the sample area, which showed a linear relationship in relation to the resulting work, and the liquid addition, which led to an abrupt decrease in adhesion, could be identified. The influence of tissue preparation was investigated in ex vivo experiments on porcine small intestinal tissue. It was found that lower values of F_max_ and W_ad_ were obtained on processed and fresh tissue than on processed and thawed tissue. Film adhesion on fresh, unprocessed tissue was lowest in most of the animals tested. Comparison of ex vivo measurements on porcine small intestinal tissue with in vitro measurements on agar/mucin gels illustrates the inter- and intra-individual variability of biological tissue.

## 1. Introduction

The use of mucoadhesive polymers in the development of modern and innovative drug delivery systems is a common formulation step [1,2,3]. In this context, mucoadhesive polymers are used for various reasons. Particularly in the case of active ingredients that are poorly permeable, such mucoadhesive dosage forms can provide a high local concentration of active ingredient on the mucosa, which should lead to improved absorption [4,5]. Furthermore, mucoadhesive dosage forms are particularly interesting for active ingredients with a small absorption window in the upper small intestine. Suitably placed, such dosage forms can improve oral bioavailability through prolonged and consistent release [6,7,8]. However, local drug therapy can also be achieved by mucoadhesive dosage forms [9].

Many different mucoadhesive dosage forms, such as tablets, films, pellets, or even semi-solid preparations, such as gels, have already been developed with different targets [10,11]. For example, mucoadhesive films can be placed locally in the esophagus to treat esophageal diseases using the EsoCap platform technology [12]. Local drug therapy may also be of interest in the stomach, for example, due to Helicobacter pylori infection [13,14]. Srivastava et al., have developed a microparticulate system for this purpose, which consists of the mucoadhesive polymer thiolated polyacrylic acid and the two active ingredients famotidine and clarithromycin [13]. Another example are mucoadhesive matrix tablets for the therapy of ulcers, which contain the mucoadhesive polymers polyacrylic acid (Carbopol) and hypromellose (HPMC) in addition to the active ingredient ranitidine hydrochloride [15]. Intestinal mucoadhesive dosage forms for the delivery of protein drugs such as insulin [16,17,18] may also benefit from increased absorption due to a local high drug concentration. A wide variety of delivery forms, such as mucoadhesive nanoparticles [16,19] or mucoadhesive patches [17,20] are the subject of research in this field.

Mucoadhesion is a complex phenomenon, and six theories are currently accepted to describe it [10,21]. These include electronic, wetting, adsorption, diffusion, mechanical, and fracture theory. The electronic theory assumes adhesion due to the formation of an electron double layer. In contrast, the adsorption theory assumes adhesion due to weak secondary bonds such as hydrogen or van der Waals bonds. The mechanic theory describes an adhesion to a rough surface by interlocking. The diffusion theory assumes that interaction between the mucus and the dosage form occurs through penetration and interaction of polymer chains of the dosage form and the mucin chains of the mucus. The wetting theory applies to liquids and describes adhesion as a function of surface and interfacial energies. Another approach is the fracture theory, which addresses the detachment force required to separate two surfaces after adhesion [21].

The extent of mucoadhesion depends on a lot of factors. On the one hand, mucoadhesion depends on the properties of the polymers, such as charge [22,23], mobility of the polymer chains, water absorption capacity, the extent of cross-linking, and molecular weight [24]. Furthermore, application site-specific factors such as the pH of the application site and the associated charge of the mucus, the presence and viscosity of wetting fluid such as saliva, gastric juice, or the amount of mucus, and mechanical stress on the dosage form at the application site [24] also influence mucoadhesion. In addition, there is high in vivo variability resulting from the particular site of application. Therefore, to estimate the mucoadhesion in vivo, biorelevant in vitro test systems can be highly interesting, especially in early development phases of innovative dosage forms or for the screening of different formulations. Various methods are reported in the literature, which can be divided into indirect and direct methods [25,26]. Indirect methods for determining adhesiveness include measuring parameters that provide information about the interactions of the mucoadhesive and the mucosa or mucus. These include, for example, rheological measurements, spectroscopic methods (e.g., Attenuated Total Reflection Fourier Transform Infrared (ATR-FTIR) spectroscopy, Nuclear Magnetic Resonance (NMR)), and determination of surface energy by contact angle measurements [27]. On the other hand, direct methods include measuring the adhesion or residence time of the dosage form on the mucosa, biomimetic gel, or a mucin compact.

Another parameter that belongs to the direct measurement methods is the force that has to be applied to separate the dosage form from the mucosa. These measurements are mostly performed as tensile assays on texture analyzers or modified microbalances. During the measurement, the mucoadhesive dosage form and the mucosa or a mucosa-mimetic material are pressed together with a defined contact force. After a defined contact time, the two surfaces are separated from each other at a specified withdrawal speed. The force-distance diagrams recorded during this process provide information on the peak force, which is defined as the maximum detachment force F_max_, and on the Area Under the Curve (AUC), which represents the work of adhesion W_ad_. A brief look at the extensive literature reviews on mucoadhesion measurements shows that many different parameters within each measurement method could potentially influence the measurement result. Factors such as the contact force and time, as well as the withdrawal speed, are test equipment-specific variables that can be freely chosen by the respective authors [26,28,29,30]. There is also a wide variety in the choice of mucosa or mucosa-mimetic materials. Animal tissues such as porcine, rat, or bovine mucosa are often used to replicate human tissue [28]. Since the results obtained on animal tissue have poor reproducibility, there are alternative approaches [31,32,33,34,35]. These include a wide variety of gels (e.g., gelatin, agar/mucin gels, HEMA-AGA hydrogels), as well as pressed mucin discs, mucin dispersions, or mucin-soaked filters [28]. In addition, the animal tissues are prepared in different ways: Some authors separated the mucosa from the underlying muscle layer before starting the experiment [36,37], others did not use fresh but thawed tissue samples, and still, other authors processed the tissues before use [38]. The tissue samples are often wetted with a defined amount of liquid before starting the experiment to better reproduce the physiological conditions at the application site. Depending on the tissue type, this can be, for example, simple buffer systems such as phosphate buffer pH 7.4 [17], but also more biorelevant wetting fluids such as Simulated Intestinal Fluid [39] or Simulated Saliva [40,41]. The simple wetting of the preparation can be further extended by a temperature-controlled measuring cell, where the mucoadhesive dosage form meets the mucosa in a humid and temperature-controlled environment [42,43,44].

Studies by Ivarsson et al., on the differences between ellipsometry, tensile strength, and rheology showed that these methods result in different conclusions regarding mucoadhesion when using the same polymers [45]. Therefore, the authors concluded that special attention must be paid to in vitro methods when comparing mucoadhesive dosage forms, as these do not lead to comparable results. However, even when using a single measurement method, such as tensile assays on the texture analyzer, the use of different measurement parameters [36,46] as well as different tissue types of different test animals [44] leads to different results, as various papers show.

These differences in the results show that it is important to comprehensively work out a measurement method for determining mucoadhesion. This includes the evaluation of device-specific parameters and sample-specific parameters. In the case of sample-specific parameters, the storage and wetting of in vitro gels and the treatment and storage of tissue in ex vivo experiments can have a decisive influence. To our knowledge, there has been no systematic investigation of different influencing parameters that includes both in vitro and ex vivo studies. In this work influencing factors such as sample area, contact force, contact time, and withdrawal speed on the adhesion of polyvinyl alcohol (PVA) films to biomimetic agar/mucin gels were investigated. In addition, the wetting of the gels and storage were to be investigated as gel-specific influencing factors. With the help of the results of these in vitro investigations, an optimized set of parameters for ex vivo measurements of small intestinal tissue of pigs was to be established. Furthermore, the effect that pretreatment of tissue has on the results of the mucoadhesion measurements was investigated in the ex vivo experiments.

## 2. Materials and Methods

### 2.1. Materials

The polymer polyvinyl alcohol EMPROVE^®^ ESSENTIAL PVA 18-88 (Merck KGaA, Darmstadt, Germany) was used to prepare the films. Anhydrous glycerol (AppliChem GmbH, Darmstadt, Germany) served as plasticizer. Demineralized water was used as solvent.

Potassium dihydrogen phosphate (neoFroxx GmbH, Einhausen, Germany), sodium hydroxide (AppliChem GmbH, Darmstadt, Germany) and deionized water were used to prepare the phosphate buffer pH 7.4 USP. Both chemicals were used in analytical grade.

Agar for microbiology (Sigma-Aldrich Chemie GmbH, Steinheim, Germany) and mucin 75–95% for biochemistry (Carl Roth GmbH & Co. KG, Karlsruhe, Germany) as well as demineralized water were used to prepare biomimetic gels.

For testing mucoadhesion on porcine intestine, porcine small intestine was obtained from a local slaughterhouse (female, 12–15 weeks of age, 35–50 kg, *n* = 3). The small intestine was examined after collection in the unprocessed and processed state. The preparation of the tissue is explained in more detail in Section 2.2.3 Preparation of animal tissue. Furthermore, prepared tissue sections were additionally deep-frozen at −20 °C, thawed for the measurements and subsequently examined.

### 2.2. Methods

#### 2.2.1. Preparation of Mucoadhesive Films

The solvent cast method was used to prepare the films. For this purpose, 80.0 g demineralized water was suspended with 18.0 g PVA 18-88 and 2.0 g glycerol in a laboratory glass bottle at 500 rpm on a magnetic stirring plate. The mixture was then heated to 85 °C for 2 h with stirring at 100 rpm in a water bath. The stirring speed was then reduced to 50 rpm and the solution was stirred without adding heat until it had cooled down to room temperature. The solution was decanted into falcon tubes and centrifuged at 4400 rpm for 15 min at room temperature to remove air bubbles (Centrifuge 5702 R, Eppendorf SE, Hamburg, Germany). The films were cast at 12.0 mm/s on a polyamide-coated liner (POLY SILK 111/105, Loparex Deutschland GmbH & Co. KG, Forchheim, Germany) with a coating knife (mtv messtechnik oHG, Erftstadt, Germany) adjusted to 1000 μm on a coating bench (Automatic Precision Film Applicator CX4, mtv messtechnik oHG, Erftstadt, Germany). After drying at room temperature for 12 h, the films were stored in airtight aluminum multilayer bags (Ströbel GmbH, Langenzenn, Germany) until further use after one week. Film thickness was measured using a mechanical thickness gauge (*n* = 10, J15, Käfer Messuhrenfabrik GmbH & Co. KG, Villingen-Schwenningen, Germany) and residual moisture was determined (*n* = 3) using a Moisture Analyzer (MB35, OHAUS Europe GmbH, Nänikon, Switzerland) at 105 °C.

#### 2.2.2. Preparation of Biomimetic Gels

2.0 g of agar was suspended in 94.0 g of demineralized water in a laboratory glass bottle and heated to 95 °C in a water bath on a magnetic stirring plate at 150 rpm for one hour to prepare the gels. The solution was then cooled to 55 °C and 4.0 g of mucin was added under stirring at 500 rpm. After a mixing time of 15 min, 15 mL of the mixture were transferred to Petri dishes, which were covered and cooled down to room temperature for 2 h. The gels were covered with parafilm, stored in the fridge at 5 °C, and were removed from refrigeration 60 min before use to investigate the effect of storage over time.

#### 2.2.3. Preparation of Animal Tissue

Ex vivo mucoadhesion experiments were performed on porcine intestinal tissue. The tissue was removed immediately after slaughter and transported stored on ice. Investigations including transport were made at least within 2 h after slaughter. Since the effects of processing the tissue on mucoadhesion were to be investigated, the tissue was divided into three sections of approximately 7 cm each. The intestinal tube was cut longitudinally, resulting in sections of approximately 5 cm × 7 cm. One third was examined without processing, one third was carefully cleaned with deionized water to remove possible food particles, and the remaining third was cleaned with deionized water and frozen. The cleaned sections intended for freezing, were packed in sealable low-density polyethylene bags (Druckverschlussbeutel LDPE transparent, packpack.de GmbH, Jever, Germany) and frozen at −20 °C in the freezer for seven days. The samples were thawed in the polyethylene bags in a water bath with constant stirring at 37 °C for 2 h.

#### 2.2.4. Adhesion Measurements with the Texture Analyzer

The investigations consisted of three main points:

- Investigation of the influence of the test equipment parameters on the maximum detachment force as well as the work of adhesion on agar/mucin gels;
- Characterization of further test parameters such as the area of the film used, wetting the gels with phosphate buffer pH 7.4 and storage of the agar/mucin gels;
- Comparative measurements of porcine intestinal tissue.

##### Influence of Instrument Parameters

**Figure 1:**
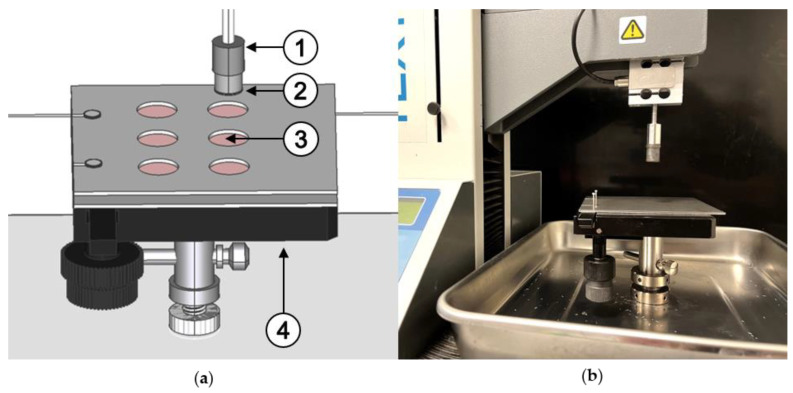
(**a**) Schematic illustration of the structural modifications made on the texture analyzer. 1: probe, 2: film sample, 3: porcine small intestine tissue and 4: microscope stage. (**b**) Photograph of the device setup in the laboratory.

Circular pieces with 14 mm diameter (A ≈ 153.94 mm^2^) were punched out of the dried films using a punching iron to measure the adhesion of the prepared films to the respective surface. These were attached to the probe of a texture analyzer (TA Plus, LLOYD Instruments, Bognor Regis, UK) using double-sided adhesive tape (tesa^®^ Doppelseitiges Klebeband universal, tesa SE, Norderstedt, Germany). Either biomimetic gels of agar and mucin or porcine small intestinal tissue were placed on the lower base. Instead of the commercially available stationary base, a microscope stage was converted to allow the most efficient use of samples, especially the tissue samples (Figure 1). The converted microscope stage allows the test substrate to be moved along x and y directions so that the specimen only needs to be placed once on the lower base at the start of the experiment. A distance of 5 cm between the film and the respective substrate was set at the beginning of each measurement. The texture analyzer was equipped with a 10 N load cell.

**Figure 2:**
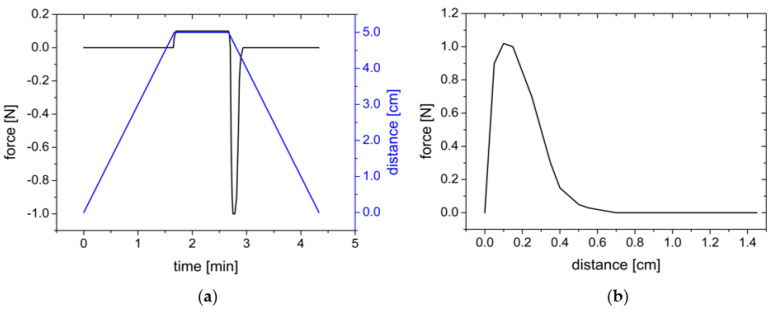
(**a**) Schematic force−time−distance diagram of the upper probe movement. (**b**) Resulting force distance diagram.

The measurement followed the same routine: The probe with the film to be tested moved at a constant speed (0.5 mm/s) towards the lower base with the test substrate. If a counterforce of 0.10 N was measured, the upper probe remained in this position for 60 s and then moved back to the starting position at a withdrawal speed of 0.5 mm/s (Figure 2). The time sequence of the movement is shown as an example in the force-time-displacement diagram in Figure 2. During the upward movement when the film was pulled off of the test substrate, a force-distance diagram was recorded, from which the maximum detachment force F_max_ and the work of adhesion W_ad_ are evaluated as the area under the curve for the evaluation.

Biomimetic gels were used to investigate the influence of test equipment parameters. The following parameters were varied starting from a standard setting: The contact force (f1–f5), the contact time of the film to the substrate (t1–t7), and the withdrawal speed (w1–w5), which are listed in Table 1.

##### Influence of Further Test Parameters

Furthermore, film sections of different sizes were applied to agar/mucin gels. The circular areas d1–d3 had a diameter of d1 = 7 mm, d2 = 10 mm and d3 = 14 mm, resulting in sample areas of A1 ≈ 38.48 mm^2^, A2 ≈ 78.54 mm^2^ and A3 ≈ 153.94 mm^2^. The influence of different amounts of phosphate buffer pH 7.4 USP (T = 22.5 ± 1.0 °C) as wetting liquid (l1–l4) was also investigated. The gels were additionally stored at 5 °C in the refrigerator for a certain time (s1–s4) after preparation to investigate the effect of storage conditions on adhesion.

The detailed measurement parameters for the measurements on the agar/mucin gels can be found in Table 1.

##### Comparative Measurements on Porcine Tissue

In addition to the investigation of test equipment parameters, the influence of tissue preparation and storage was also characterized. For this purpose, measurements were performed at measurement parameters resulting from the initial investigations on biomimetic agar/mucin gels. The reader is referred to Section 3.1.6. for the description of these measurement conditions.

During the measurements, the tissues were additionally weighted with a perforated plate made of stainless steel to prevent the tissue from lifting off when force was applied. For all tests performed on the texture analyzer, the number of samples was *n* = 6. 1000 data points were recorded in each case. The maximum detachment force F_max_ was evaluated as the peak force during detachment and the work of adhesion W_ad_ as the area under the force-distance diagram.

## 3. Results and Discussion

### 3.1. Influence of Test Equipment Parameters on the Adhesiveness of Films on Biomimetic Gels

In order to evaluate the optimal measurement parameters for subsequent investigations of the tissues, experiments were carried out with biomimetic gels based on agar and mucin. The PVA films used for this purpose had an average film thickness of 128 (±1.5) μm and residual moisture of 10.48 (±3.2) % in the dried state.

#### 3.1.1. Influence of the Sample Area

**Figure 3:**
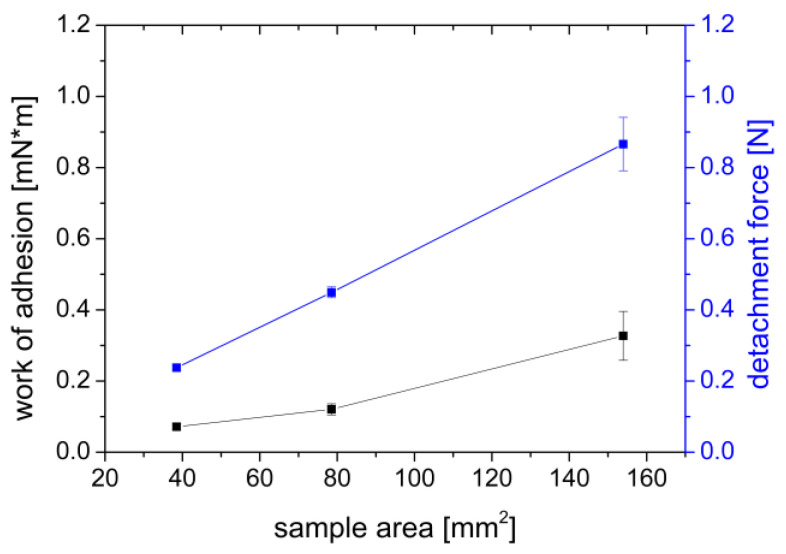
Effect of sample area of a PVA film on the work of adhesion (mN×m) and the detachment force (N) measured on a gel of 2% agar and 4% mucin. Mean ± SD, *n* = 6.

With increasing film sample area, the adhesion force increased from 0.234 N for a sample area of A = 38.48 mm^2^ to 0.866 N for a sample area of A = 153.94 mm^2^, and the work of adhesion increased from 0.072–0.327 mN×m (Figure 3). As expected, the adhesion force and work increased approximately linear with the area of the film used. The linear relationship between the sample area and the measured values can be confirmed by the regression coefficient of R^2^(F_max_) = 0.9996 and R^2^(W_ad_) = 0.8164. An approximately linear relationship between the sample area with the adhesion work and the maximum detachment force has also been observed by Göbel et al. [47] when they investigated the influence of the sample area of circular HPMC and PVA films with a diameter of 5–20 mm on adhesion to gelatin type A gels.

#### 3.1.2. Influence of the Contact Force

**Figure 4:**
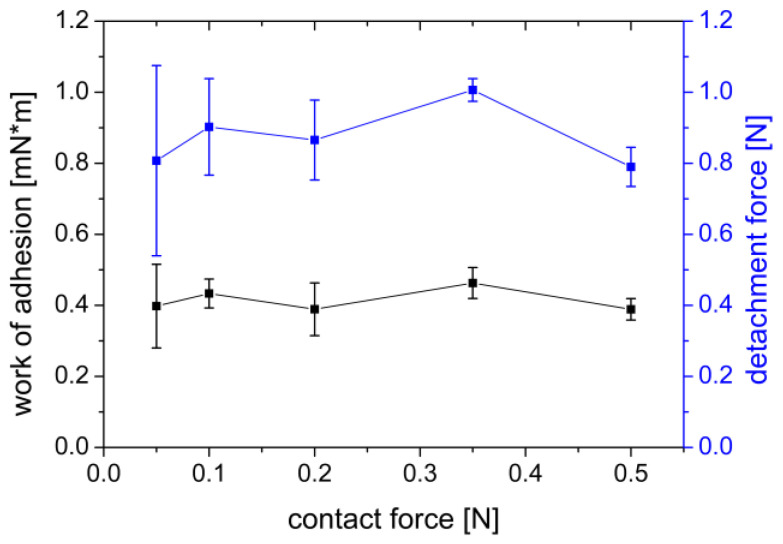
Effect of contact force of a PVA film on the work of adhesion (mN×m) and the detachment force (N) measured on a gel of 2% agar and 4% mucin. Mean ± SD, *n* = 6.

A range of 0.05–0.50 N was selected to investigate the influence of adhesion contact force. The results shown in Figure 4, point out that the work of adhesion measured at constant parameters varied from 0.389–0.463 mN×m, as did the adhesion force, which ranged from 0.790–1.010 N. A trend of the measured values over the changed parameter of the contact force could not be observed. Macroscopic observation of the gels also showed no defects due to a damaged gel structure in any case. However, it can be seen from the standard deviations that the variability of the measured values decreases with increasing contact pressure. The lower variability could be related to a higher precision of the load cell at higher forces.

Comparable observations were also made by Wong et al. [48] when they examined mucoadhesive tablets of Carbopol 974P and Methocel K4M on chicken pouch. The authors did not observe any significant effect of contact force in the range of 0.05–0.10 N and from 0.5–1.0 N on adhesion at low contact times below one minute. For contact times exceeding one minute, a statistically significant difference was only observed in percentage more extensive of contact force from 0.05–0.50 N. Adhesion requires intimate contact of the dosage form to the respective substrate (tissue or biomimetic gel). The more completely the dosage form rests, the better it can interact with the substrate. At the same time, however, the contact force must not be so high that it could damage the tissue.

#### 3.1.3. Influence of Contact Time

**Figure 5:**
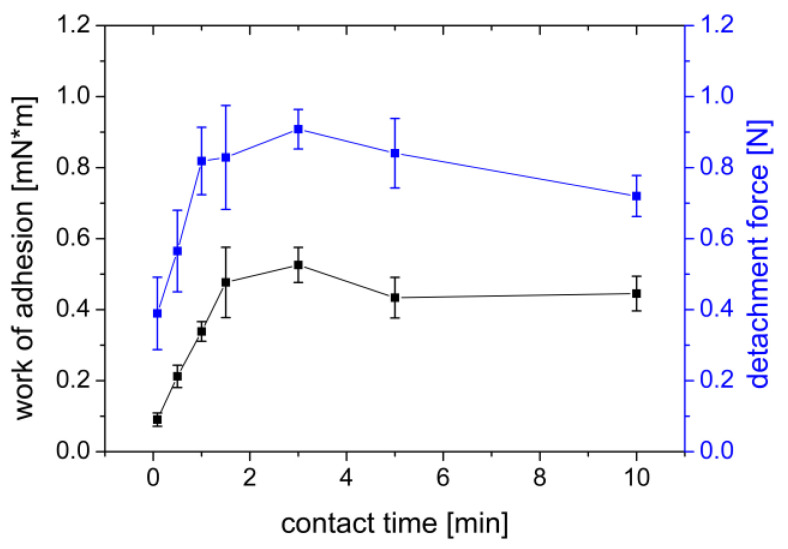
Influence of contact time of a PVA film on work of adhesion (mN×m) and detachment force (N) measured on a gel of 2% agar and 4% mucin. Mean ± SD, *n* = 6.

When measuring the influence of the contact time of the film on the gel, times in the range of 5–600 s were chosen. After 180 s of contact time, a maximum was observed in the measured work (W_ad_ = 0.5261 mN×m) and the detachment force (F_max_ = 0.9083 N) (Figure 5). With longer contact time, the measured values decreased again, but not as fast as they increased. After a contact time of 600 s, the measured work decreased to W_ad_ = 0.4451 mN×m and the detachment force decreased to F_max_ = 0.7201 N.

These observations may be explained by the PVA’s chemical structure and the mucoadhesion diffusion theory. PVA is a nonionic polymer that swells slowly in water. Due to this slower swelling, it takes time for the polymer chains to be able to diffusely interact with the mucin chains of the agar/mucin gel. As the swelling of the PVA progresses, however, adhesion again decreases as the previously dry and hydrophilic film decomposes over time to a gel-like structure that adheres more poorly to the agar/mucin gel. Göbel et al. [47] investigated the influence of the contact time of PVA films on gelatin type A gels in vitro. They could not observe a clear trend in the observed time from 3–120 s of contact time. With a longer contact time as in our study, a trend could have possibly been observed.

Solid dosage forms tend to break at the contact surface when the adhesive bond is released from a surface, as this is where the weakest bond is found. In gel-like preparations, however, the weakest bond is often found within the gel, so that the mucoadhesive dosage form tears when detached from the respective test substrate [36].

Estrellas et al. [49] studied the adhesion of different polymers to small pieces of intestinal tissue ex vivo and obtained similar results. They concluded that hydrophilic polymers such as polycarbophil, which can swell rapidly, adhere to tissue quickly but also lose their bioadhesion to rat small intestinal tissue quickly. In contrast, polymers with more hydrophobic backbones exhibit higher adhesion. However, transferring their results to our in vitro study is problematic due to the different experimental conditions. In the study of Estrellas et al., different amounts of liquid can be assumed on the small intestine tissue than on the biomimetic agar/mucin gels. The amount of fluid may affect swelling and, thus, mucoadhesion of the PVA polymer. Preconditioning of the biomimetic gels with wetting fluids could be performed to reproduce these conditions in in vitro experiments.

#### 3.1.4. Influence of the Withdrawal Speed

**Figure 6:**
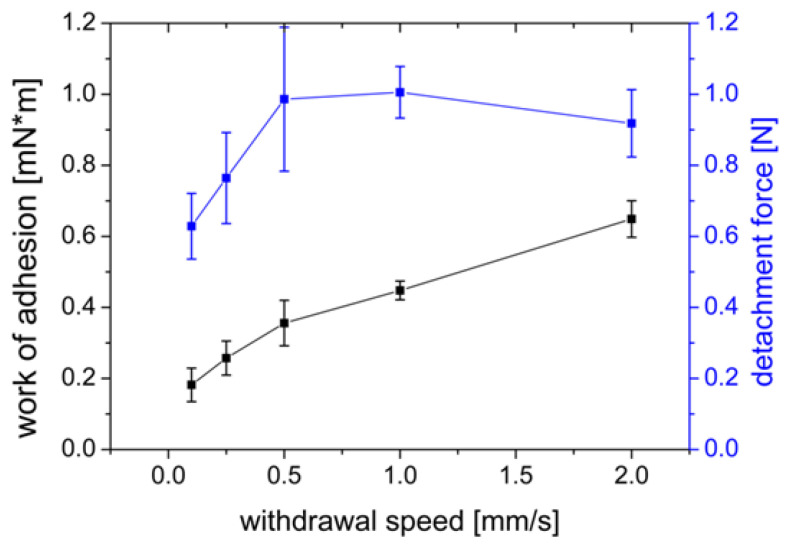
Influence of the withdrawal speed of a PVA film on the work of adhesion (mN×m) and detachment force (N) measured on a gel of 2% agar and 4% mucin. Mean ± SD, *n* = 6.

When varying the withdrawal speed of the PVA film from the agar/mucin gel, it can be observed that the work of adhesion increased with increasing withdrawal speed (Figure 6). The maximum detachment force was observed with a value of F_max_ = 1.006 N at a withdrawal speed of 1.0 mm/s and decreased again with further increasing withdrawal speed. In experiments with chicken pouch and Carbopol 974P and Methocel K4M tablets, Wong et al. [48] observed an overall increase in the work of adhesion and maximum detachment force the faster the specimen was removed from the tissue.

The withdrawal speeds studied in their work ranged from 0.1 to 1.0 mm/s. A slight decrease in the maximum detachment force from 0.5 mm/s to 1.0 mm/s was observed for the tablets made of Carbopol 974P. In our study, the maximum detachment force decreases from a velocity of 1.0 mm/s. These differences may be because the polymers are different and the tissue used by Wong et al. [48] was wetted, unlike the biomimetic agar/mucin gels.

A slow withdrawal speed may decrease adhesion as there may be a lack of dissipation in the gel [50]. In their study, Baus et al. [41] investigated withdrawal speeds from 0.1–2.0 mm/s and concluded that both F_max_ and W_ad_ are lower at lower withdrawal speeds. The lower forces and reduced work are thought to be caused by the elastic properties of the gel that occur when the film contacts the respective substrate [36]. Due to higher stress, which results from higher withdrawal speeds, the time for bond deformation is reduced, resulting in higher measurable adhesion [51].

#### 3.1.5. Addition of Wetting Liquid

**Figure 7:**
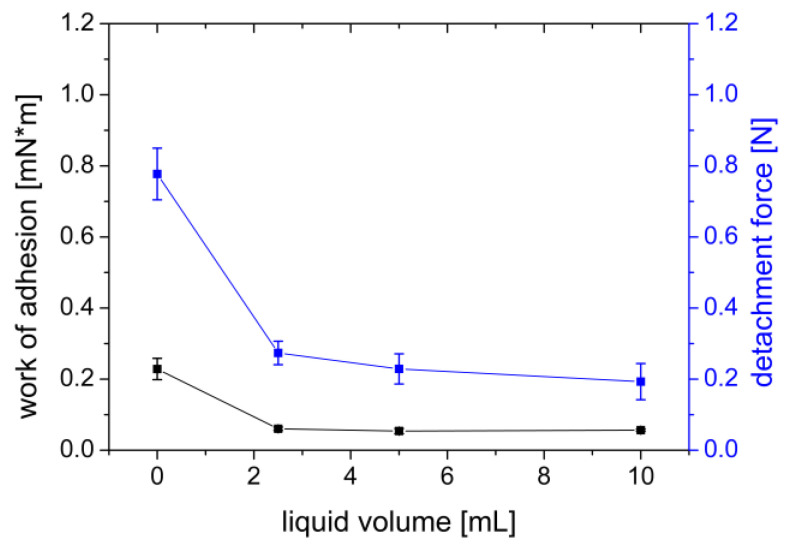
Effect of adding wetting liquid (PBS buffer pH 7.4) of a gel of 2% agar and 4% mucin on work of adhesion (mN×m) and detachment force (N) measured with a PVA film. Mean ± SD, *n* = 6.

Adding even small amounts (2.5 mL) of phosphate buffer pH 7.4 USP to a freshly prepared agar/mucin gel abruptly decreased the adhesion of the PVA film from F_max_ = 0.777 N and W_ad_ = 0.229 mN×m to F_max_ = 0.274 N and W_ad_ = 0.061 mN×m (Figure 7).

One reason for better adhesion to drier surfaces could be the movement of water from the mucus layer into the film as described by Baus et al. [41]. Due to this osmotic effect, the PVA film adheres well to comparatively dry surfaces. As a nonionic and hydrophilic polymer, the PVA mainly binds to the agar/mucin gel through secondary bonds. These secondary bonds are mostly hydrogen bonds. When a wetting liquid is added, the hydrogen bonds preferentially interact with it rather than with the underlying agar/mucin gel. Moreover, the additional presence of solvent can lead to faster swelling of the PVA film. The consequences of faster swelling have already been discussed in Section 3.1.3.

#### 3.1.6. Influence of Gel Storage

**Figure 8:**
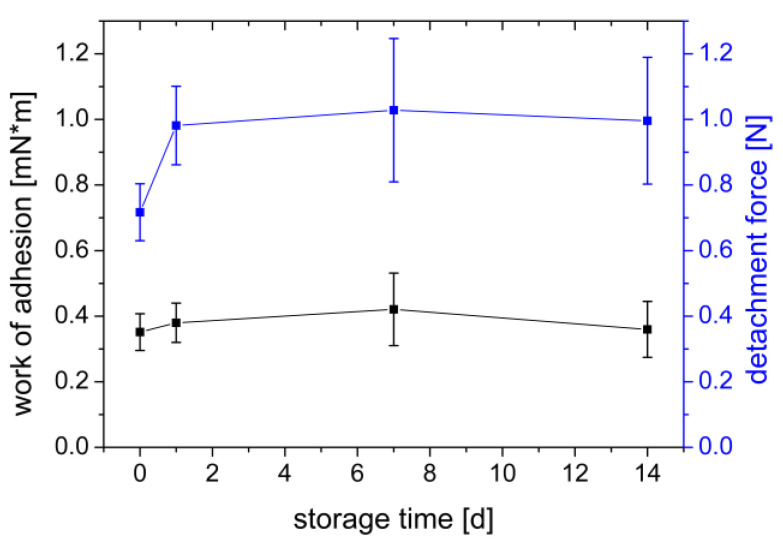
Influence of storage time of a gel of 2% agar and 4% mucin on work of adhesion (mN×m) and detachment force (N) measured with a PVA film. Mean ± SD, *n* = 6.

When investigating the effect of storage time on the adhesion, it is clear that there was a change in the adhesion of the PVA films to the gel surface over time (Figure 8). On the production day, the measured work of adhesion was lowest (0.352 mN×m), increased with time until day 7 (0.421 mN×m), and decreased back to the initial level after 14 days of storage (0.356 mN×m). However, the influence of the storage time is more pronounced in terms of the maximum detachment force. The detachment force increased from the day of manufacture (0.717 N) to the following day (0.981 N) and remained essentially constant after (1.028–0.996 N). Agar gels are known to be subject to the phenomenon of syneresis. This involves spontaneous shrinkage of the gel and separation of the bound water [52]. The water collects in tiny droplets on the surface of the gel. At the same time, there is an increase in the concentration of the polymers in the gel. The increase in mucin concentration due to water separation during storage is possibly the reason for the increased adhesion of the PVA film. With increasing concentration of a mucoadhesive polymer, more polymer chains are available for crosslinking with the mucin chains, resulting in greater adhesion [24]. Similarly, it is also conceivable that increasing mucin concentration, increases adhesion as more mucin chains can interact.

The experiments to assess adhesion using PVA films and agar/mucin gels showed that using a larger sample area can lead to stronger adhesion. At the same time, adding a wetting liquid can reduce the adhesion of a PVA film. Furthermore, the storage time of biomimetic gels also plays a role. Therefore, they should always be freshly prepared before starting the experiment to obtain reproducible results.

Of the test equipment-specific parameters, the contact force has the most negligible influence on the adhesion of the films to the biomimetic gels. The contact time, on the other hand, influences the adhesion to the extent that when using 128 (±1.5) μm thick PVA films, a maximum can be observed after a time of 180 s for both the measured maximum detachment force and the calculated W_ad_. For the withdrawal speed, it can be observed that the work of adhesion increases with increasing withdrawal speed, while the tear-off force is at its maximum at 1 mm/s.

From the results of the tests on the agar-mucin gels, it was possible to derive an optimized test equipment parameter, which is favoring the highest possible detachment force F_max_ and work of adhesion W_ad_ of PVA films on the agar/mucin gels. For example, the contact force was adjusted to include the structural makeup of the tissue used below. The small intestinal tissue of pigs is partially compressible. Higher contact forces could compress the tissue more, with the tissue losing integrity as a result [48]. At the same time, the sample should adhere as completely as possible to the tissue, which is textured relative to the gel, because adhesion is usually higher on smooth surfaces than on uneven surfaces [53]. A higher contact force and a resulting relaxation of the tissue can lead to a smoothing of the surface of the tissue. The influence of the contact force does not seem to have a great impact on the measurement results in the measured range, which is why a force of 0.35 N was chosen due to the minimization of damage to the tissues. The optimum contact time of the PVA film for the highest possible tear-off force and WoA was 180 s. The influence of the withdrawal speed on the tear-off force has its optimum at 1.0 mm/s, while the influence on the WoA increases further with increasing withdrawal speed. In favor of a controlled film peeling, a withdrawal speed of 1.0 mm/s was chosen for the optimized measurement conditions. The diameter of the circular PVA film section was also 14 mm (A ≈ 154 mm^2^) for the adhesion measurements on porcine small intestinal tissue.

### 3.2. Comparative Measurements on Porcine Small Intestine Tissue

To investigate the influence of small intestine tissue processing on mucoadhesion, tissue from three pigs was measured using the optimized test equipment parameters described previously. Three measurements were performed for each experimental animal:

- First, the tissue was used in fresh and uncleaned condition;
- Second series of measurements were performed on fresh, cleaned tissue;
- Third series of measurements consisted of cleaned tissue frozen for seven days.

**Figure 9:**
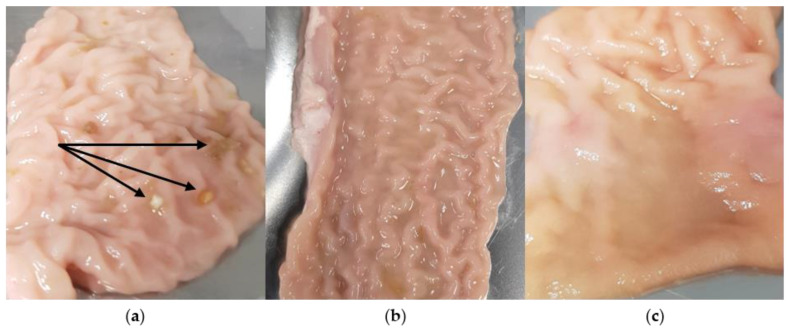
Differences between the individual tissue samples of the pig small intestines. (**a**) pig #1; (**b**) pig #2; (**c**) pig #3. The intestinal tube was cut longitudinally and divided into approx. 5 cm × 7 cm sections. Food components (about 3 mm) present in pig #1 are marked with arrows.

As shown in Figure 9, visible differences were already evident between the samples of the individual test animals. While in pig #1, food components adhered to the mucosa, only liquid components were visible in pig #2 and pig #3.

Since the test animals had food and water available before slaughter and tissue removal, this explains the different filling states of the small intestine. On the one hand, this can lead to poor interindividual comparability and reproducibility [31]. In vivo, fluids are always present in the small intestine regardless of the prandial state. In the fasted state, a constant secretion of mucus as well as small amounts of gastric acid, bile and pancreatic juice can be expected. In the fed state, the number of components emptied from the stomach increases, as does the production of bile and pancreatic juice [54]. During careful cleansing of the tissues, these solid and liquid components can be removed. This suggests that the uncleaned intestine may better represent the fed state in vivo.

**Figure 10:**
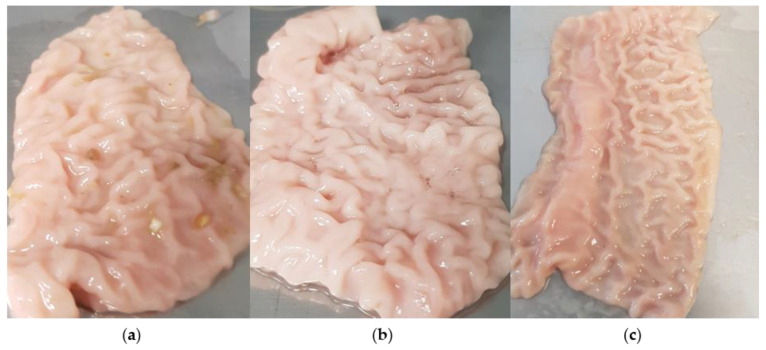
Intraindividual differences between processed tissue samples from pig #1. (**a**) Unprocessed tissue; (**b**) cleaned tissue; (**c**) thawed tissue.

The effects of processing on the tissue were also macroscopically visible as exemplified by the tissue of pig #1 in Figure 10. While individual food components are still visible on the fresh, unprocessed tissue, they are no longer visible on the section of the small intestine washed with deionized water. The thawed washed tissue lost notable firmness during thawing, and the folding (plicae circularis) was less pronounced. In addition, tissue fluid leaked during the thawing of the tissue samples.

The fact that the structure of the mucus layer of the porcine nasal mucosa is also changed during thawing was also observed by Hägerström et al. [36]. They concluded that the tissue should be used as fresh as possible. During the thawing of the specimens, there was a leakage of tissue fluid. This can be attributed to the presence of ice crystals. Ice crystals are formed when tissue is frozen without antifreeze agents [55]. Depending on the speed of the freezing process, these ice crystals may damage the cells by perforation. In addition, ice formation can lead to osmotic processes, which in turn can damage the integrity of the tissue [55]. Baraibar et al., observed in studies on canine small intestines that there was an increase in autolysis of the mucosa during freezing for seven days and subsequent thawing. They also observed that no mucus was detectable after thawing.

**Figure 11:**
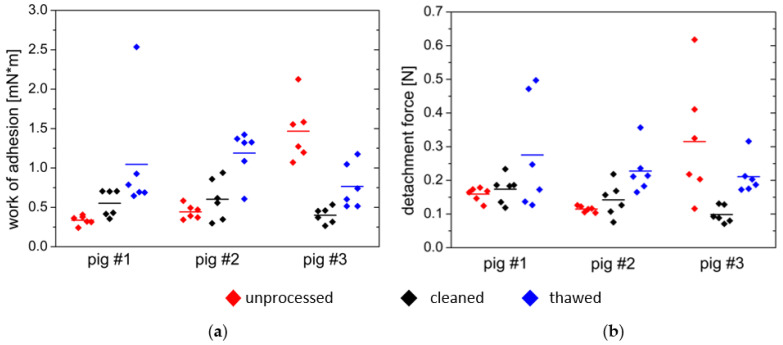
(**a**) Work of adhesion in mN×m and (**b**) maximum detachment force in N required to detach PVA films from differently prepared tissue samples. Shown are the individual data and median, *n* = 6.

The effect of these intra- and interindividual differences can not only be seen macroscopically but also in the mucoadhesion measurements. Figure 11 shows that the maximum detachment force F_max_ and the work of adhesion W_ad_ differ between individuals and interindividual with tissue processing.

A trend can be seen in the tissue samples from pig #1 and pig #2, as can be seen in Figure 11. The work of adhesion and maximum detachment force were both low on the uncleaned tissue and showed a low variation of measured values. The thawed, cleaned tissue showed a higher adhesion work and maximum detachment force in both cases. The variation of the measured values was also higher within one test animal. In pig #3, the uncleaned tissue differed from the observations of the measurements of pig #1 and pig #2. The adhesion appeared to be very high on this tissue, which is illustrated by a mean work of adhesion of W_ad_ = 1.467 mN×m and a mean maximum detachment force of F_max_ = 0.292 N in contrast to W_ad_ = 0.402 mN×m and F_max_ = 0.098 N for the cleaned tissue. At the same time, the variation of the measured values was high for the fresh, uncleaned fabric.

The most notable interindividual differences could be found with a value range of approximately W_ad_ = 0.3–1.5 mN×m in the fresh, uncleaned tissue (Table 2). For the fresh, cleaned tissue, the work of adhesion values ranged from W_ad_ = 0.4 mN×m to W_ad_ = 0.6 mN×m and were thus closer to each other. Adhesion on thawed tissue ranged from W_ad_ = 0.77 mN×m to W_ad_ = 1.2 mN×m.

Adhesion can be influenced by a wide variety of parameters that differ interindividual and are challenging to evaluate. Especially the amount of fluid can influence mucoadhesion. This can vary depending on the filling state of the small intestine. Additionally, the thickness and viscosity of the mucus layer can influence mucoadhesion as shown by Varum et al. [37]. The authors observed that to detach a pellet of Carbopol 974P NF from gastric mucosa, a significantly higher adhesion work W_ad_ had to be applied than when detaching mucosa from the jejunum. As a possible reason, the different thickness of the respective mucus layer is mentioned, which in pigs is significantly thicker in the stomach (about 51–68 μm) than in the jejunum (about 29 μm). Due to the thicker mucus layer, a more substantial and deeper chain diffusion could take place, which leads to stronger adhesion, according to the authors.

When carefully cleaning the tissue samples, damage to the mucus layer may occur. Depending on the cleaning intensity, there may be dilution or even washing away of the mucus. From this consideration, one would expect adhesion to be lower after cleaning. In the present results, this was the case only in one of three samples (pig #3). The amount of liquid after washing may be lower in pig #1 and pig #2 than in the uncleaned state, which could result in higher adhesion. In contrast, Mortazavi and Smart observed in their studies that the presence of mucus decreased the adhesive forces [56]. The thickness and texture of the mucosa could have been determined using histological sections to conclude the effect of cleaning and thawing on the mucosa. This should be part of further investigations. Since mucoadhesion is a highly complex process that can be influenced by many factors, some of which are also mutually dependent, the reasons for the observations can only be speculated.

Comparing the measurement results on the tissues with the mucosa-mimicking agar/mucin gels (Table 2), it is noticeable that the maximum detachment force of 1.104 N is much higher for the gel than for the tissues (0.098–0.292 N). For the work of adhesion, the value for the gels of 0.730 mN×m is in the range of 0.337–1.467 N measured on the tissue. Because the variability of the tissue is so high, the agar/mucin gel used can only replicate mucoadhesion to tissue to a limited extent. For example, gels for the different prepared tissues could be investigated in further studies to represent a worst-case and a best-case of adhesion.

When characterizing and deriving the measurement parameters considered optimal, it should be noted that they do not have general validity but represent the optimal measurement conditions for PVA films on agar/mucin gels in the texture analyzer. Other parameters may be necessary depending on the dosage form or polymer used. For polymers that swell quickly, a shorter contact time may be necessary. On the other hand, solid dosage forms such as tablets sometimes require more time to absorb and swell water from the mucosa-mimicking gel. In these cases, wetting the gel with liquid or preswelling the dosage form may be beneficial to simulate physiological conditions [37]. Thus, many variables should be considered in mucoadhesion measurement.

## 4. Conclusions

The presented study successfully characterized a measurement method for determining the mucoadhesion of PVA films. Various factors affect the measurement results when measuring the mucoadhesion of PVA films on agar/mucin gels. For the test equipment-specific parameters of contact force, contact time and withdrawal speed, the influence of contact time in particular was observed as an influencing variable. The optimum contact time for the PVA films investigated was 180 s. The withdrawal speed also influences the test results—the adhesion work increases with a higher withdrawal speed. Less influence on the measured variables was observed for the contact force. The sample area, the wetting liquid’s presence, and the gels’ age were identified as further influencing variables.

An optimized set of parameters was derived from the experiments on the mucosa-mimicking agar/mucin gels, which were used to perform ex vivo experiments on small intestinal tissue from pigs. In the ex vivo experiments, the effect of tissue preparation was investigated. Intraindividual differences were found depending on whether the tissue was used in the uncleaned fresh state, cleaned fresh state, or cleaned and thawed state. In two out of three pigs, an increase in the maximum detachment force F_max_ and the work of adhesion W_ad_ could be observed from the uncleaned fresh to the cleaned fresh and the cleaned thawed tissue. Notable interindividual variability could also be observed. In addition to reducing the use of experimental animals, the findings obtained in this study also highlight the need for mucosa-mimicking gels with high reproducibility of results. Ideally, these should be able to cover a wide range of tissue types to represent interindividual differences as well. Further studies should be performed on human tissues to investigate the comparability of mucosa-mimicking gels, potential animal tissues used ex vivo, and human tissues. In particular, human tissues suggest even more pronounced interindividual variability due to age, sex, and potential disease of the tissue.

## Figures and Tables

**Table 1:** Measurement parameters of the in vitro experiments as well as the ex vivo experiments.

Sample	Settings	Contact Force	Contact Time	Withdrawal Speed	Sample Area	Liquid	Storage Time	Processing
		N	s	mm/s	mm^2^	mL	d	
gel	standard	0.10	60	0.50	153.94	-	-	-
	f1	0.05	60	0.50	153.94	-	-	-
	f2	0.10						
	f3	0.20						
	f4	0.35						
	f5	0.50						
	t1	0.10	5	0.50	153.94	-	-	-
	t2		30					
	t3		60					
	t4		90					
	t5		180					
	t6		300					
	t7		600					
	w1	0.10	60	0.10	153.94	-	-	-
	w2			0.25				
	w3			0.50				
	w4			1.00				
	w5			2.00				
	a1	0.10	60	0.50	38.48	-	-	-
	a2				78.54			
	a3				153.94			
	l1	0.10	60	0.50	153.94	0.00	-	-
	l2					2.50		
	l3					5.00		
	l4					10.00		
	s1	0.10	60	0.50	153.94	-	-	-
	s2						1	
	s3						7	
	s4						14	
	optimized *	0.35	180	1.00	153.94	-	-	-
tissue	optimized1 *	0.35	180	1.00	153.94	-	-	unprocessed
	optimized2 *						-	cleaned
	optimized3 *						7	thawed

* Optimized settings resulted from studies conducted on agar/mucin gels which were carried out to investigate influencing parameters. For detailed information on the origin, the reader is referred to Section 3.1.6.

**Table 2:** Overview of the results (mean ± SD) of the mucoadhesion in vitro and ex vivo tests at the test parameters Fcontact = 0.35 N; tcontact = 180 s and withdrawal = 1.0 mm/s.

		W_ad_	F_max_
		mN×m	N
pig #1	unprocessed	0.337 ± 0.058	0.147 ± 0.020
	cleaned	0.553 ± 0.168	0.144 ± 0.041
	Thawed	1.046 ± 0.737	0.217 ± 0.168
pig #2	unprocessed	0.443 ± 0.090	0.111 ± 0.009
	cleaned	0.599 ± 0.260	0.121 ± 0.050
	thawed	1.190 ± 0.307	0.190 ± 0.068
pig #3	unprocessed	1.467 ± 0.380	0.292 ± 0.180
	cleaned	0.402 ± 0.101	0.098 ± 0.025
	thawed	0.766 ± 0.283	0.165 ± 0.054
agar/mucin gel		0.730 ± 0.122	1.104 ± 0.060

## Data Availability

The data presented in this study are available on request from the corresponding author.
